# Supplementary material for: Visual Processing Within and Across Hemispheres: A Systems Factorial Technology Analysis
Source: Comput Brain Behav. 2025 Jun 2;8(4):607–48. doi: 10.1007/s42113-025-00249-5 (PMC13298663; doi:10.1007/s42113-025-00249-5)
Supplement: Supplementary file 1 — Supplementary file1 (DOCX 809 KB) [file 42113_2025_249_MOESM1_ESM.docx]

**Supplemental Material**

In this supplemental material includes two categories of items: i) tables containing the number of correct trials in all of the conditions for Experiments 1 and 2, and ii) additional analysis done on the centrally presented Navon arrow stimuli from Experiment 1, for observer A1 and A2. The additional analysis is the same as what was presented in the manuscript: survivor function (both single- and redundant-target), survivor interaction contrasts (SICs), mean interaction contrasts (MICs), and capacity coefficient analyses. We present similar tables and figures with our analysis and conclusions from that analysis.

**Tables A1 and A2**

**Table A1.** Correct trials for all observers in all conditions used for SFT modeling – arrow stimuli

| Observer | Task | Visual Field | Condition | Trials |
| --- | --- | --- | --- | --- |
| A1 | OR | LVF | AA | 164 |
|  |  |  | AL | 117 |
|  |  |  | AH | 119 |
|  |  |  | LA | 111 |
|  |  |  | HA | 124 |
|  |  |  | HH | 56 |
|  |  |  | HL | 59 |
|  |  |  | LH | 59 |
|  |  |  | LL | 58 |
|  |  | RVF | AA | 212 |
|  |  |  | AL | 113 |
|  |  |  | AH | 117 |
|  |  |  | LA | 112 |
|  |  |  | HA | 121 |
|  |  |  | HH | 58 |
|  |  |  | HL | 60 |
|  |  |  | LH | 57 |
|  |  |  | LL | 59 |
|  |  | Bi | AA | 85 |
|  |  |  | AL | 114 |
|  |  |  | AH | 117 |
|  |  |  | LA | 111 |
|  |  |  | HA | 119 |
|  |  |  | HH | 59 |
|  |  |  | HL | 58 |
|  |  |  | LH | 59 |
|  |  |  | LL | 60 |
|  | AND | LVF | AA | 234 |
|  |  |  | AL | 122 |
|  |  |  | AH | 117 |
|  |  |  | LA | 117 |
|  |  |  | HA | 117 |
|  |  |  | HH | 57 |
|  |  |  | HL | 59 |
|  |  |  | LH | 56 |
|  |  |  | LL | 50 |
|  |  | RVF | AA | 240 |
|  |  |  | AL | 119 |
|  |  |  | AH | 117 |
|  |  |  | LA | 116 |
|  |  |  | HA | 122 |
|  |  |  | HH | 61 |
|  |  |  | HL | 57 |
|  |  |  | LH | 62 |
|  |  |  | LL | 50 |
|  |  | Bi | AA | 239 |
|  |  |  | AL | 119 |
|  |  |  | AH | 115 |
|  |  |  | LA | 115 |
|  |  |  | HA | 114 |
|  |  |  | HH | 56 |
|  |  |  | HL | 60 |
|  |  |  | LH | 59 |
|  |  |  | LL | 52 |
| A2 | OR | LVF | AA | 211 |
|  |  |  | AL | 115 |
|  |  |  | AH | 123 |
|  |  |  | LA | 76 |
|  |  |  | HA | 92 |
|  |  |  | HH | 58 |
|  |  |  | HL | 61 |
|  |  |  | LH | 61 |
|  |  |  | LL | 61 |
|  |  | RVF | AA | 241 |
|  |  |  | AL | 119 |
|  |  |  | AH | 112 |
|  |  |  | LA | 117 |
|  |  |  | HA | 110 |
|  |  |  | HH | 60 |
|  |  |  | HL | 56 |
|  |  |  | LH | 22 |
|  |  |  | LL | 24 |
|  |  | Bi | AA | 187 |
|  |  |  | AL | 118 |
|  |  |  | AH | 121 |
|  |  |  | LA | 78 |
|  |  |  | HA | 98 |
|  |  |  | HH | 62 |
|  |  |  | HL | 60 |
|  |  |  | LH | 56 |
|  |  |  | LL | 61 |
|  | AND | LVF | AA | 249 |
|  |  |  | AL | 117 |
|  |  |  | AH | 116 |
|  |  |  | LA | 112 |
|  |  |  | HA | 112 |
|  |  |  | HH | 52 |
|  |  |  | HL | 54 |
|  |  |  | LH | 19 |
|  |  |  | LL | 18 |
|  |  | RVF | AA | 202 |
|  |  |  | AL | 117 |
|  |  |  | AH | 117 |
|  |  |  | LA | 82 |
|  |  |  | HA | 101 |
|  |  |  | HH | 63 |
|  |  |  | HL | 57 |
|  |  |  | LH | 59 |
|  |  |  | LL | 57 |
|  |  | Bi | AA | 235 |
|  |  |  | AL | 116 |
|  |  |  | AH | 113 |
|  |  |  | LA | 116 |
|  |  |  | HA | 112 |
|  |  |  | HH | 51 |
|  |  |  | HL | 59 |
|  |  |  | LH | 29 |
|  |  |  | LL | 15 |

**Table A2.** Correct trials for all observers in all conditions used for SFT modeling – Gabor patches

| Participant | Task | Visual Field | Condition | Trials |
| --- | --- | --- | --- | --- |
| G1 | OR | LVF | AA | 878 |
|  |  |  | AL | 217 |
|  |  |  | AH | 220 |
|  |  |  | LA | 188 |
|  |  |  | HA | 224 |
|  |  |  | HH | 56 |
|  |  |  | HL | 55 |
|  |  |  | LH | 54 |
|  |  |  | LL | 56 |
|  |  | RVF | AA | 874 |
|  |  |  | AL | 218 |
|  |  |  | AH | 223 |
|  |  |  | LA | 201 |
|  |  |  | HA | 223 |
|  |  |  | HH | 56 |
|  |  |  | HL | 56 |
|  |  |  | LH | 56 |
|  |  |  | LL | 56 |
|  |  | Bi | AA | 1008 |
|  |  |  | AL | 254 |
|  |  |  | AH | 256 |
|  |  |  | LA | 238 |
|  |  |  | HA | 255 |
|  |  |  | HH | 64 |
|  |  |  | HL | 64 |
|  |  |  | LH | 56 |
|  |  |  | LL | 56 |
|  | AND | LVF | AA | 239 |
|  |  |  | AL | 219 |
|  |  |  | AH | 212 |
|  |  |  | LA | 236 |
|  |  |  | HA | 237 |
|  |  |  | HH | 317 |
|  |  |  | HL | 279 |
|  |  |  | LH | 102 |
|  |  |  | LL | 186 |
|  |  | RVF | AA | 237 |
|  |  |  | AL | 207 |
|  |  |  | AH | 205 |
|  |  |  | LA | 232 |
|  |  |  | HA | 227 |
|  |  |  | HH | 317 |
|  |  |  | HL | 267 |
|  |  |  | LH | 150 |
|  |  |  | LL | 216 |
|  |  | Bi | AA | 236 |
|  |  |  | AL | 223 |
|  |  |  | AH | 219 |
|  |  |  | LA | 235 |
|  |  |  | HA | 238 |
|  |  |  | HH | 318 |
|  |  |  | HL | 272 |
|  |  |  | LH | 114 |
|  |  |  | LL | 234 |
| G2 | OR | LVF | AA | 973 |
|  |  |  | AL | 250 |
|  |  |  | AH | 251 |
|  |  |  | LA | 237 |
|  |  |  | HA | 251 |
|  |  |  | HH | 63 |
|  |  |  | HL | 64 |
|  |  |  | LH | 62 |
|  |  |  | LL | 62 |
|  |  | RVF | AA | 985 |
|  |  |  | AL | 244 |
|  |  |  | AH | 253 |
|  |  |  | LA | 233 |
|  |  |  | HA | 254 |
|  |  |  | HH | 63 |
|  |  |  | HL | 64 |
|  |  |  | LH | 64 |
|  |  |  | LL | 62 |
|  |  | Bi | AA | 975 |
|  |  |  | AL | 255 |
|  |  |  | AH | 254 |
|  |  |  | LA | 250 |
|  |  |  | HA | 254 |
|  |  |  | HH | 64 |
|  |  |  | HL | 64 |
|  |  |  | LH | 63 |
|  |  |  | LL | 64 |
|  | AND | LVF | AA | 236 |
|  |  |  | AL | 191 |
|  |  |  | AH | 216 |
|  |  |  | LA | 233 |
|  |  |  | HA | 234 |
|  |  |  | HH | 319 |
|  |  |  | HL | 303 |
|  |  |  | LH | 172 |
|  |  |  | LL | 288 |
|  |  | RVF | AA | 237 |
|  |  |  | AL | 188 |
|  |  |  | AH | 225 |
|  |  |  | LA | 229 |
|  |  |  | HA | 230 |
|  |  |  | HH | 318 |
|  |  |  | HL | 308 |
|  |  |  | LH | 269 |
|  |  |  | LL | 306 |
|  |  | Bi | AA | 235 |
|  |  |  | AL | 198 |
|  |  |  | AH | 228 |
|  |  |  | LA | 231 |
|  |  |  | HA | 233 |
|  |  |  | HH | 317 |
|  |  |  | HL | 311 |
|  |  |  | LH | 249 |
|  |  |  | LL | 300 |
| G3 | OR | LVF | AA | 973 |
|  |  |  | AL | 237 |
|  |  |  | AH | 255 |
|  |  |  | LA | 204 |
|  |  |  | HA | 253 |
|  |  |  | HH | 63 |
|  |  |  | HL | 64 |
|  |  |  | LH | 64 |
|  |  |  | LL | 61 |
|  |  | RVF | AA | 987 |
|  |  |  | AL | 250 |
|  |  |  | AH | 252 |
|  |  |  | LA | 246 |
|  |  |  | HA | 253 |
|  |  |  | HH | 64 |
|  |  |  | HL | 62 |
|  |  |  | LH | 64 |
|  |  |  | LL | 62 |
|  |  | Bi | AA | 975 |
|  |  |  | AL | 252 |
|  |  |  | AH | 251 |
|  |  |  | LA | 242 |
|  |  |  | HA | 256 |
|  |  |  | HH | 64 |
|  |  |  | HL | 64 |
|  |  |  | LH | 63 |
|  |  |  | LL | 62 |
| G4 | OR | LVF | AA | 1006 |
|  |  |  | AL | 234 |
|  |  |  | AH | 249 |
|  |  |  | LA | 231 |
|  |  |  | HA | 249 |
|  |  |  | HH | 63 |
|  |  |  | HL | 63 |
|  |  |  | LH | 63 |
|  |  |  | LL | 62 |
|  |  | RVF | AA | 1006 |
|  |  |  | AL | 248 |
|  |  |  | AH | 254 |
|  |  |  | LA | 246 |
|  |  |  | HA | 251 |
|  |  |  | HH | 64 |
|  |  |  | HL | 62 |
|  |  |  | LH | 63 |
|  |  |  | LL | 61 |
|  |  | Bi | AA | 1010 |
|  |  |  | AL | 249 |
|  |  |  | AH | 254 |
|  |  |  | LA | 251 |
|  |  |  | HA | 254 |
|  |  |  | HH | 64 |
|  |  |  | HL | 64 |
|  |  |  | LH | 63 |
|  |  |  | LL | 63 |

**Analyses of Central Field Condition, Exp. 1**

**Figure S1.** Single-target condition survivor functions for the hierarchical arrow stimuli, “OR” task (central presentation)

Observer A1


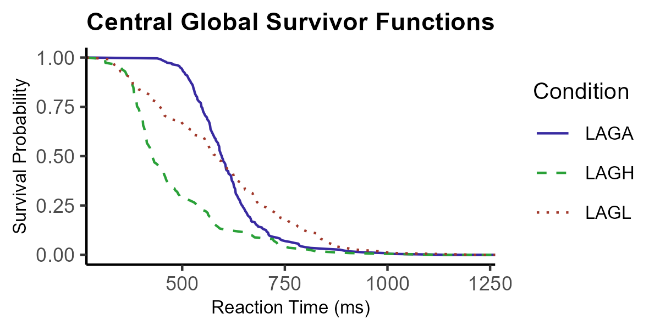

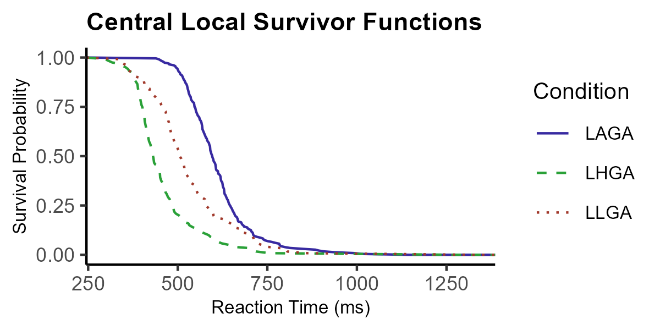


Observer A2


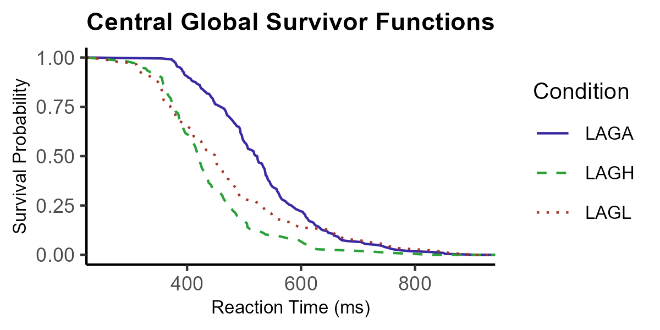

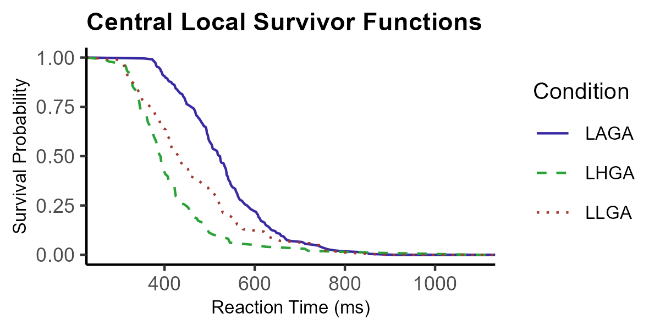


*Note.* Survivor functions for the single-target conditions across each visual field for observers A1 (top two rows) and A2 (bottom two rows) in the “OR” task. For each observer, the first column displays the distributions for the conditions where there was no target arrow on the local level but the target on the global level may or may not have been present. The second column displays the distributions for the conditions where there was no target arrow on the global level but the target on the local level may or may not have been present. LAGA = local absent, global absent; LAGH = local absent, global high; LAGL = local absent, global low; LHGA = local high, global absent; LLGA = local low, global absent.

**Figure S2.** Survivor functions, SIC curves, and MIC interaction plots for the hierarchical arrow stimuli, “OR” task (central presentation)

Observer A1


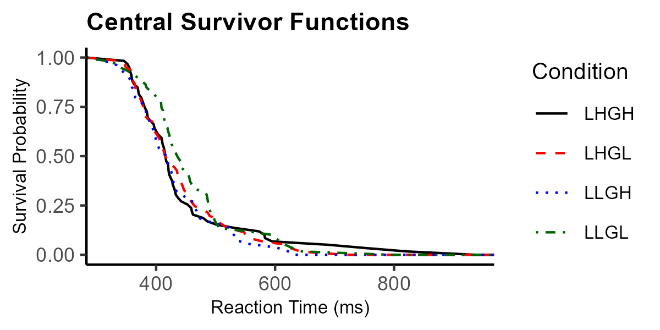

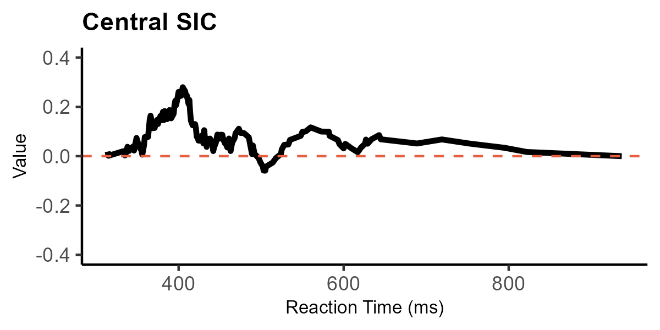


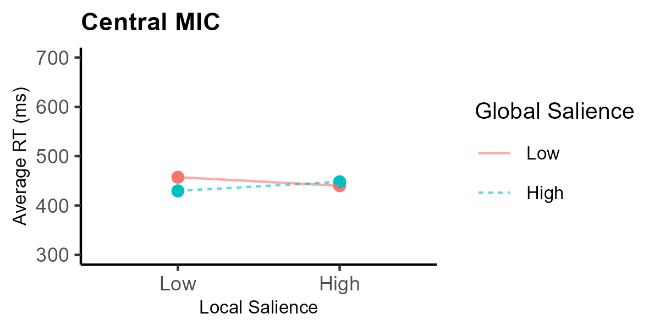


Observer A2


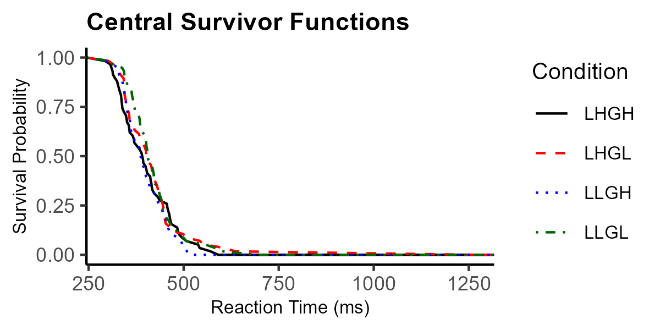

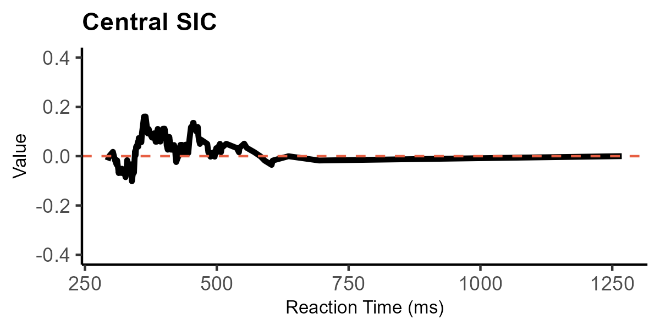


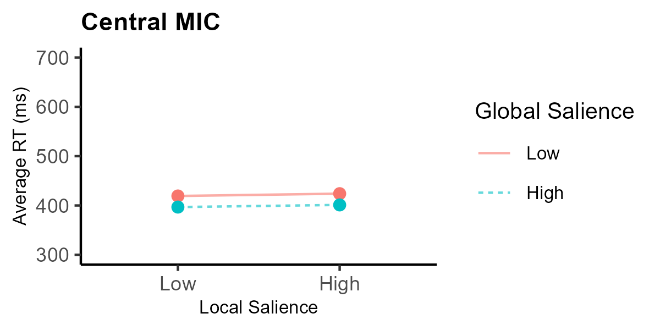


*Note.* Survivor functions, SIC curves, and MIC interaction plots for the redundant-target conditions for observers A1 (top three rows) and A2 (bottom three rows) in the “OR” task. For each observer, the first row contains the survivor functions and SIC curves (from left to right). The second row contains the MIC interaction plots. LHGH = local high, global high; LHGL = local high, global low; LLGH = local low, global high; LLGL = local low, global low; SIC = survivor interaction contrast; MIC = mean interaction contrast.

**Table S1.** Two-sample Kolmogorov-Smirnov test for the hierarchical arrow stimuli, “OR” task (central presentation) – single and redundant components

| **Observer Number** | **Single Component** | | **Redundant Component** | | | |
| --- | --- | --- | --- | --- | --- | --- |
|  | **AH vs. AL** | **HA vs. LA** | **HH < HL** | **HH < LH** | **LL > HL** | **LL > LH** |
| A1 | 0.395*** | 0.411*** | 0.138 | 0.059 | 0.206^‡^ | 0.260* |
| A2 | 0.177* | 0.241** | 0.171 | 0.138 | 0.161 | 0.224^‡^ |

*Note.* All values represent the (two-sample) Kolmogorov-Smirnov D test statistic. AH = local absent, global high; AL = local absent, global low; HA = local high, global absent; LA = local low, global absent; HH = local high, global high; HL = local high, global low; LH = local low, global high; LL = local low, global low.

*** p < 0.001; ** p < 0.01; * p < 0.05; ‡ p < 0.10.

**Table S2.** MIC and SIC results for the hierarchical arrow stimuli, “OR” task (central presentation)

| **Observer Number** | **MIC Results** | | | **SIC Results** | | |
| --- | --- | --- | --- | --- | --- | --- |
|  | **MIC** | $\boldsymbol{\eta}_{\boldsymbol{p}}^{\boldsymbol{2}}$ | **Conclusion** | **SIC > 0** | **SIC < 0** | **Conclusion** |
| A1 | 35.762 | 0.010 | zero | 0.280 | 0.059 | zero |
| A2 | − 0.344 | 0.001 | zero | 0.161 | 0.102 | zero |

*Note.* MIC = mean interaction contrast value (with significance coming from a 2 × 2 factorial ANOVA interaction effect); $\eta_{p}^{2}$ = partial-eta squared effect size for ANOVA interaction effect; SIC = survivor interaction contrast $\hat{D}^{+}$ (SIC > 0) and $\hat{D}^{-}$ (SIC < 0) test statistic value via a generalization of the (two-sample) Kolmogorov-Smirnov test statistic. zero = no significant increase or decrease from zero; positive = significant increase from zero; negative = significant decrease from zero.

**Figure S3.** Capacity coefficient functions for the hierarchical arrow stimuli, “OR” task (central presentation)

Observer A1 Observer A2


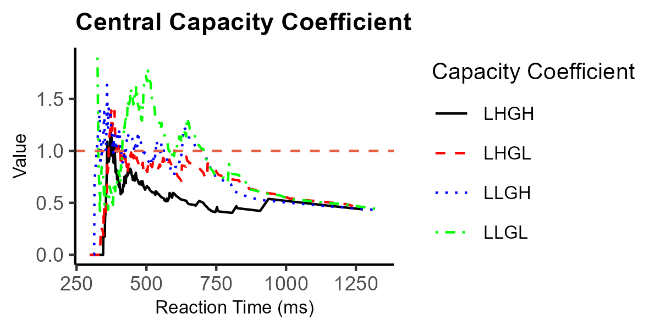

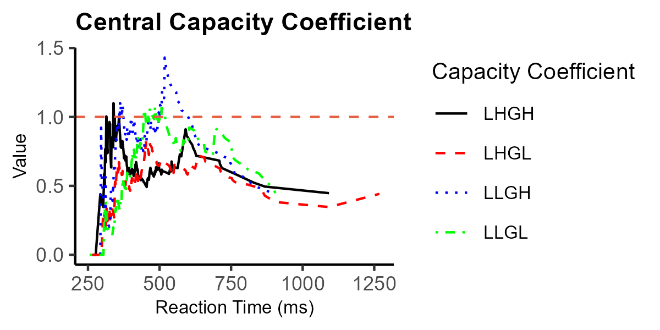


*Note.* Capacity coefficient functions unique for each redundant-contrast condition across each visual field for observers A1 (left) and A2 (right) in the “OR” task. LHGH = local high, global high; LHGL = local high, global low; LLGH = local low, global high; LLGL = local low, global high.

**Table S3.** Capacity coefficient results for hierarchical arrow stimuli, “OR” task (central presentation)

| **Observer Number** | **Capacity Results** | | | | | | | |
| --- | --- | --- | --- | --- | --- | --- | --- | --- |
|  | **LHGH** | | **LHGL** | | **LLGH** | | **LLGL** | |
|  | **Z** | **Conclusion** | **Z** | **Conclusion** | **Z** | **Conclusion** | **Z** | **Conclusion** |
| A1 | − 3.549*** | L | − 0.641 | UL | 0.301 | UL | 1.342 | UL |
| A2 | − 2.982** | L | − 3.532*** | L | − 0.258 | UL | − 1.203 | UL |

*Note*. LHGH = local high, global high; LHGL = local high, global low; LLGH = local low global high; LLGL = local low, global low. Z = Nelson-Aalen log-rank test statistic value; SC = supercapacity; UL = unlimited capacity; L = limited capacity.

*** p < 0.001; ** p < 0.01

**Figure S4.** Single-target condition survivor functions for the hierarchical arrow stimuli, “AND” task (central presentation)

Observer A1


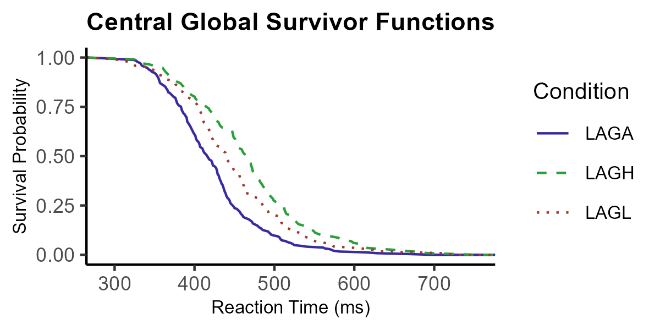

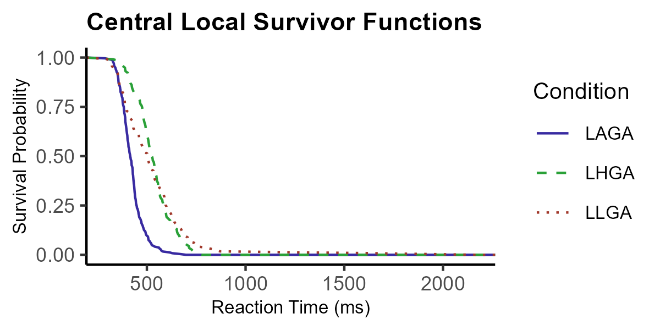


Observer A2


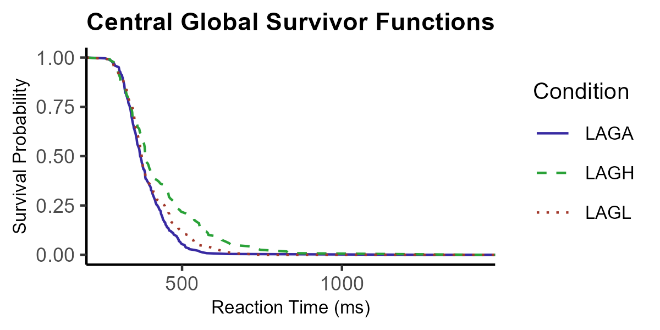

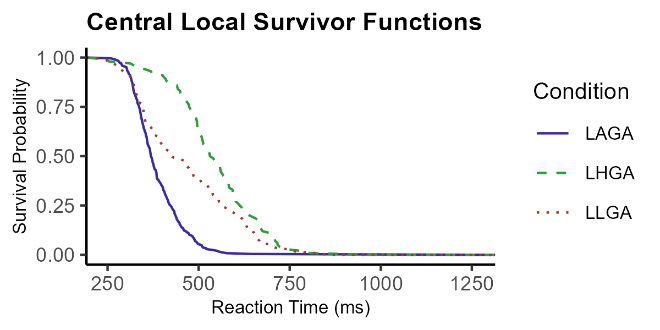


*Note.* Survivor functions for the single-target conditions across each visual field for observers A1 (top two rows) and A2 (bottom two rows) in the “AND” task. For each observer, the first column displays the distributions for the conditions where there was no target arrow on the local level but the target on the global level may or may not have been present. The second column displays the distributions for the conditions where there was no target arrow on the global level but the target on the local level may or may not have been present. LAGA = local absent, global absent; LAGH = local absent, global high; LAGL = local absent, global low; LHGA = local high, global absent; LLGA = local low, global absent.

**Figure S5.** Survivor functions, SIC curves, and MIC interaction plots for the hierarchical arrow stimuli, “AND” task (central presentation)

Observer A1


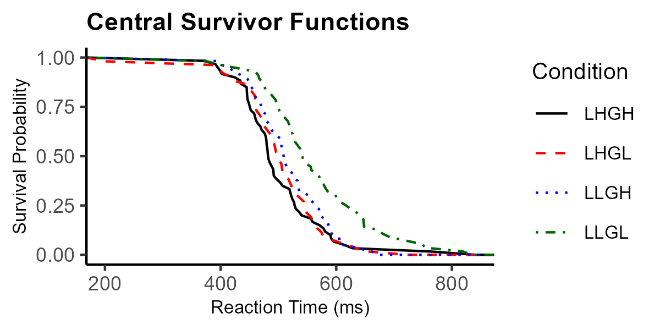

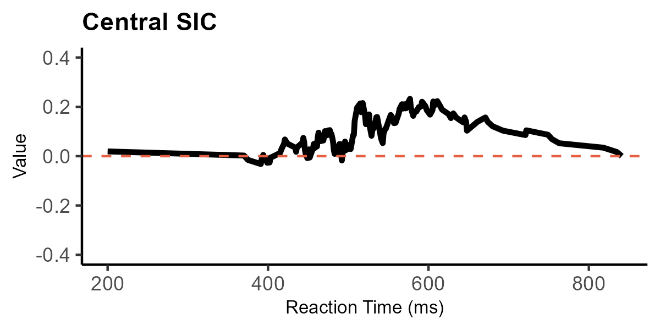


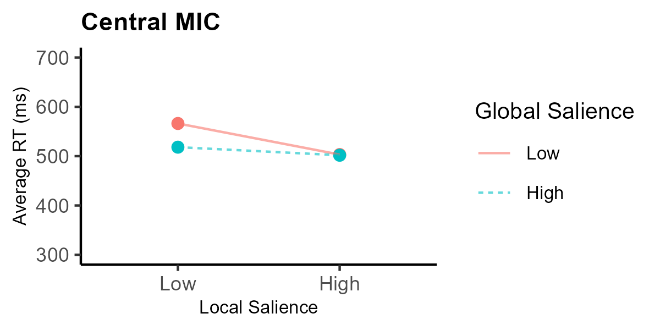


Observer A2


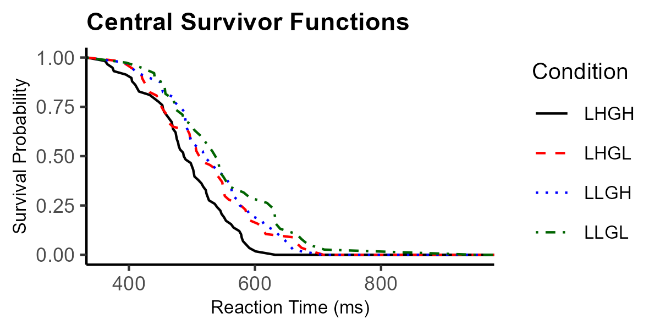

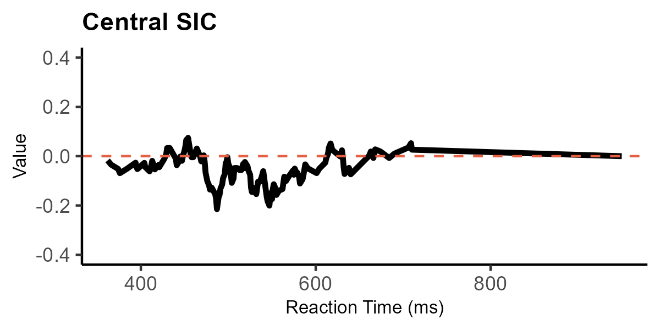


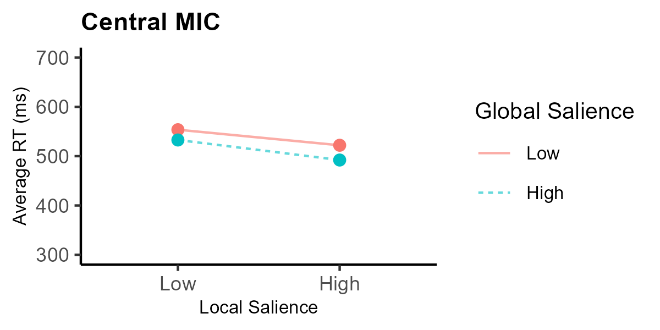


*Note.* Survivor functions, SIC curves, and MIC interaction plots for the redundant-target conditions for observers A1 (top three rows) and A2 (bottom three rows) in the “AND” task. For each observer, the first row contains the survivor functions (left) and SIC curves (right). The second row contains the MIC interaction plots. LHGH = local high, global high; LHGL = local high, global low; LLGH = local low, global high; LLGL = local low, global low; SIC = survivor interaction contrast; MIC = mean interaction contrast.

**Table S4.** Two-sample Kolmogorov-Smirnov test for the hierarchical arrow stimuli, “AND” task (central presentation) – single and redundant components

| **Observer Number** | **Single Component** | | **Redundant Component** | | | |
| --- | --- | --- | --- | --- | --- | --- |
|  | **AH vs. AL** | **HA vs. LA** | **HH < HL** | **HH < LH** | **LL > HL** | **LL > LH** |
| A1 | 0.189* | 0.209** | 0.196 | 0.241* | 0.310** | 0.268* |
| A2 | 0.142^‡^ | 0.383*** | 0.198 | 0.250^‡^ | 0.184 | 0.139 |

*Note.* All values represent the (two-sample) Kolmogorov-Smirnov D test statistic. AH = local absent, global high; AL = local absent, global low; HA = local high, global absent; LA = local low, global absent; HH = local high, global high; HL = local high, global low; LH = local low, global high; LL = local low, global low.

*** p < 0.001; ** p < 0.01; * p < 0.05; ‡ p < 0.10.

**Table S5.** MIC and SIC results for the hierarchical arrow stimuli, “AND” task (central presentation)

| **Observer Number** | **MIC Results** | | | **SIC Results** | | |
| --- | --- | --- | --- | --- | --- | --- |
|  | **MIC** | $\boldsymbol{\eta}_{\boldsymbol{p}}^{\boldsymbol{2}}$ | **Conclusion** | **SIC > 0** | **SIC < 0** | **Conclusion** |
| A1 | 46.633* | 0.021 | positive | 0.232 | 0.032 | zero |
| A2 | − 9.094 | 0.001 | zero | 0.074 | 0.215 | zero |

*Note.* MIC = mean interaction contrast value (with significance coming from a 2 × 2 factorial ANOVA interaction effect); $\eta_{p}^{2}$ = partial-eta squared effect size for ANOVA interaction effect; SIC = survivor interaction contrast $\hat{D}^{+}$ (SIC > 0) and $\hat{D}^{-}$ (SIC < 0) test statistic value via a generalization of the (two-sample) Kolmogorov-Smirnov test statistic. zero = no significant increase or decrease from zero; positive = significant increase from zero; negative = significant decrease from zero.

* p < 0.05

**Figure S6.** Capacity coefficient functions for the hierarchical arrow stimuli, “AND” task (central presentation)

Observer A1


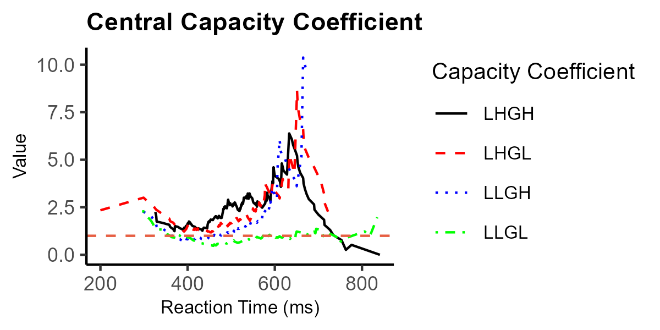

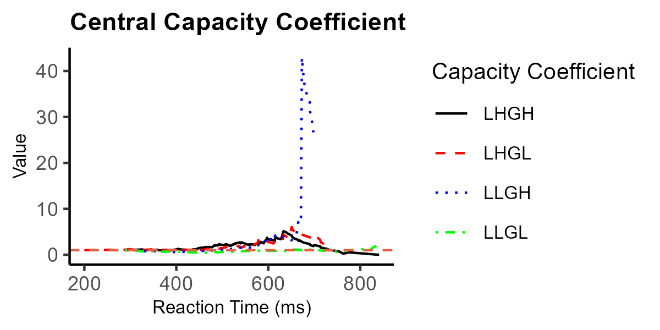


Observer A2


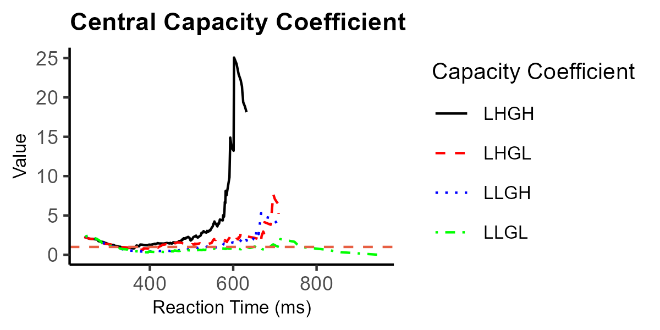

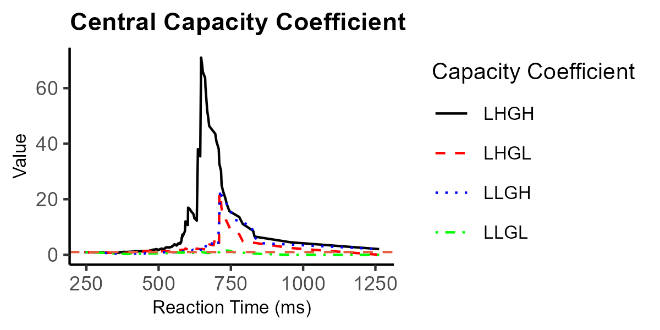


*Note.* Capacity coefficient functions unique for each redundant-contrast condition across each visual field for observers A1 (top two row) and A2 (bottom two row) in the “AND” task. For each observer, column one corresponds to the “classic” capacity calculation whereas column two corresponds to the “modified” capacity calculation. LHGH = local high, global high; LHGL = local high, global low; LLGH = local low, global high; LLGL = local low, global high.

**Table S6.** Capacity coefficient results for hierarchical arrow stimuli, “AND” task (central presentation)

| **Observer Number** | **Capacity Results** | | | | | | | |
| --- | --- | --- | --- | --- | --- | --- | --- | --- |
|  | **LHGH** | | **LHGL** | | **LLGH** | | **LLGL** | |
|  | **Z** | **Conclusion** | **Z** | **Conclusion** | **Z** | **Conclusion** | **Z** | **Conclusion** |
| A1 | 4.011*** | SC | − 0.641 | UL | 0.301 | UL | 1.342 | UL |
| A2 | 2.876** | SC | 0.983 | UL | − 2.173* | L | − 2.972** | L |

*Note*. LHGH = local high, global high; LHGL = local high, global low; LLGH = local low global high; LLGL = local low, global low. Z = Nelson-Aalen log-rank test statistic value; SC = supercapacity; UL = unlimited capacity; L = limited capacity.

*** p < 0.001; ** p < 0.01; * p < 0.05.
